# Author Correction: Increasing species sampling in chelicerate genomic-scale datasets provides support for monophyly of Acari and Arachnida

**DOI:** 10.1038/s41467-019-12259-6

**Published:** 2019-10-01

**Authors:** Jesus Lozano-Fernandez, Alastair R. Tanner, Mattia Giacomelli, Robert Carton, Jakob Vinther, Gregory D. Edgecombe, Davide Pisani

**Affiliations:** 1 University of Bristol School of Biological Sciences, 24 Tyndall Avenue, Bristol, BS8 1TQ UK; 2 University of Bristol School of Earth Sciences, 24 Tyndall Avenue, Bristol, BS8 1TQ UK; 3 Department of Biology, The National University of Ireland Maynooth, Maynooth, Kildare Ireland; 4 Department of Earth Sciences, The Natural History Museum, Cromwell Road, London, SW7 5BD UK; 5 Present Address: Department of Evolutionary Biology, Ecology and Environmental Sciences, & Biodiversity Research Institute (IRBio) Universitat de Barcelona, Avinguda Diagonal 643, Barcelona, 08028 Spain

**Keywords:** Classification and taxonomy, Phylogenetics, Phylogenomics, Biodiversity

Correction to: *Nature Communications* 10.1038/s41467-019-10244-7, published online 24 May 2019.

The original version of this Article omitted the following from the Acknowledgements:

This research was funded by a NERC BETR grant (NE/P013678/1 to D.P. and J.V.), and the European Union’s Horizon 2020 research and innovation programme under the Marie Skłodowska-Curie grant agreement (764840 to D.P. and M.G.).

This has now been corrected in both the PDF and HTML versions of the Article.

