# Supplementary material for: Increasing species sampling in chelicerate genomic-scale datasets provides support for monophyly of Acari and Arachnida
Source: Nat Commun. 2019 May 24;10:2295. doi: 10.1038/s41467-019-10244-7 (PMC6534568; doi:10.1038/s41467-019-10244-7)
Supplement: Supplementary file 1 — Supplementary Information [file 41467_2019_10244_MOESM1_ESM.pdf]

Supplementary Information

Increasing species sampling in chelicerate genomic-scale datasets provides support for monophyly of Acari and Arachnida

Lozano-Fernandez *et al.*

Table of contents:

**Supplementary Figure 1**	2
**Supplementary Figure 2**	3-4
**Supplementary Figure 3**	5-7
**Supplementary Table 1**	8-9
**Supplementary Table 2**	10-12
**Supplementary Table 3**	13-14

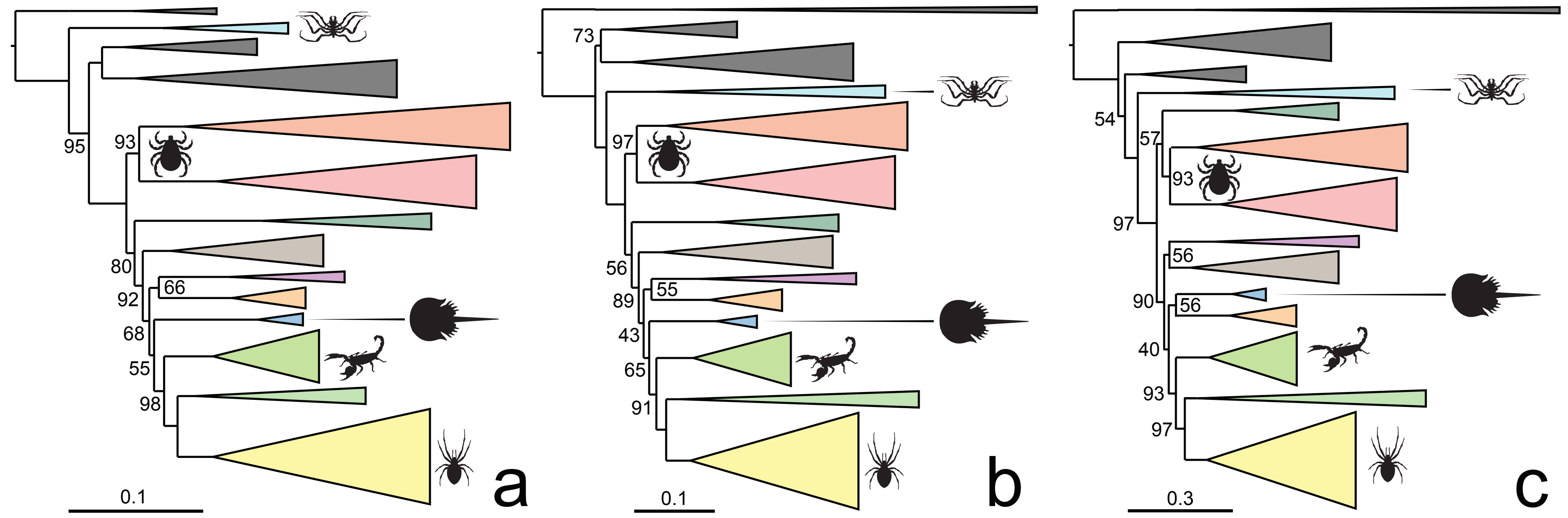

Supplementary Figure 1. Schematic representation of ML results. **(A)** Schematic representation of the relationships inferred from Matrix A using ML under the LG+I+G4 model. **(B)** Schematic representation of the relationships inferred from Matrix B using ML under the LG +I+G4 model.**(C)** Schematic representation of the relationships inferred from Matrix C using ML under the LG+F+I+G4 model. Support values represent bootstrap values, with lack of a number indicating maximum support. Silhouettes have been designed by ART.

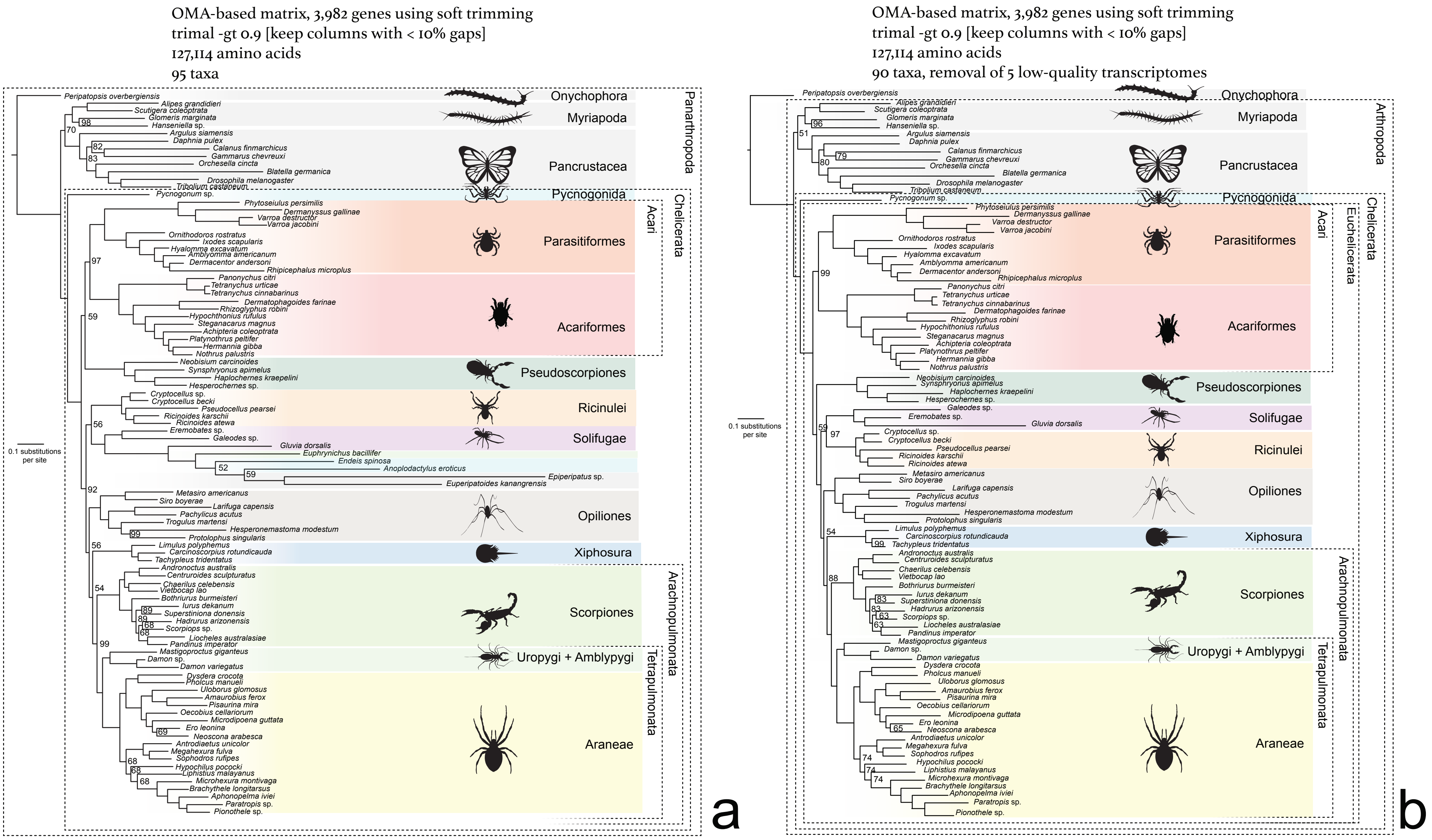

OMA-based matrix, 3,982 genes using soft trimming
trimal -gt 0.9 [keep columns with < 10% gaps]
127,114 amino acids
95 taxa
Peripatopsis overbergiensis
Alipes grandidieri
Scutigera coleoptrata
Glomeris marginata
Hanseniella sp.
Argulus siamensis
Daphnia pulex
Calanus finmarchicus
Gammarus chevreuxi
Orchesella cincta
Blatella germanica
Drosophila melanogaster
Tribolium castaneum
Pycnogonum sp.
Phytoseiulus persimilis
Dermanyssus gallinae
Varroa destructor
Varroa jacobini
Ornithodoros rostratus
Ixodes scapularis
Hyalomma excavatum
Amblyomma americanum
Dermacentor andersoni
Rhipicephalus microplus
Panonychus citri
Tetranychus urticae
Tetranychus cinnabarinus
Dermatophagoides farinae
Rhizoglyphus robini
Hypochthonius rufulus
Steganacarus magnus
Achipteria coleoptrata
Platynothrus peltifer
Hermannia gibba
Nothrus palustris
Neobisium carcinoides
Synsphyronus apimelus
Haplochernes kraepelini
Hesperochernes sp.
Cryptocellus sp.
Cryptocellus becki
Pseudocellus pearsei
Ricinoides karschii
Ricinoides atewa
Eremobates sp.
Galeodes sp.
Gluvia dorsalis
Euphrynichus bacillifer
Endeis spinosa
Anoplodactylus eroticus
Epiperipatus sp.
Euperipatoides kanangrensis
Metasiro americanus
Siro boyerae
Larifuga capensis
Pachylicus acutus
Trogulus martensi
Hesperonemastoma modestum
Protolophus singularis
Limulus polyphemus
Carcinoscorpius rotundicauda
Tachypleus tridentatus
Andronoctus australis
Centruroides sculpturatus
Chaerilus celebensis
Vietbocap lao
Bothriurus burmeisteri
Iurus dekanum
Superstitionia donensis
Hadrurus arizonensis
Scorpiops sp.
Liocheles australasiae
Pandinus imperator
Mastigoproctus giganteus
Damon sp.
Damon variegatus
Dysdera crocota
Pholcus manueli
Uloborus glomosus
Amaurobius ferox
Pisaurina mira
Oecobius cellariorum
Microdipoena guttata
Ero leonina
Neoscona arabesca
Antrodiaetus unicolor
Megahexura fulva
Sophodros rufipes
Hypochilus pococki
Liphistius malayanus
Microhexura montivaga
Brachythele longitarsus
Aphonopelma iviei
Paratropis sp.
Pionothele sp.
70
98
82
83
97
59
56
52
59
92
99
56
54
89
89
68
68
99
69
68
68
68
0.1 substitutions per site
Onychophora
Myriapoda
Pancrustacea
Pycnogonida
Parasitiformes
Acariformes
Pseudoscorpiones
Ricinulei
Solifugae
Opiliones
Xiphosura
Scorpiones
Uropygi + Amblypygi
Araneae
Panarthropoda
Chelicerata
Acari
Arachnopulmonata
Tetrapulmonata
a
OMA-based matrix, 3,982 genes using soft trimming
trimal -gt 0.9 [keep columns with < 10% gaps]
127,114 amino acids
90 taxa, removal of 5 low-quality transcriptomes
Peripatopsis overbergiensis
Alipes grandidieri
Scutigera coleoptrata
Glomeris marginata
Hanseniella sp.
Argulus siamensis
Daphnia pulex
Calanus finmarchicus
Gammarus chevreuxi
Orchesella cincta
Blatella germanica
Drosophila melanogaster
Tribolium castaneum
Pycnogonum sp.
Phytoseiulus persimilis
Dermanyssus gallinae
Varroa destructor
Varroa jacobini
Ornithodoros rostratus
Ixodes scapularis
Hyalomma excavatum
Amblyomma americanum
Dermacentor andersoni
Rhipicephalus microplus
Panonychus citri
Tetranychus urticae
Tetranychus cinnabarinus
Dermatophagoides farinae
Rhizoglyphus robini
Hypochithonius rufulus
Steganacarus magnus
Achipteria coleoptrata
Platynothrus peltifer
Hermannia gibba
Nothrus palustris
Neobisium carcinoides
Synsphryonus apimelus
Haplochernes kraepelini
Hesperochernes sp.
Galeodes sp.
Eremobates sp.
Gluvia dorsalis
Cryptocellus sp.
Cryptocellus becki
Pseudocellus pearsei
Ricinoides karschii
Ricinoides atewa
Metasiro americanus
Siro boyerae
Larifuga capensis
Pachylicus acutus
Trogulus martensi
Hesperonemastoma modestum
Protolophus singularis
Limulus polyphemus
Carcinoscorpius rotundicauda
Tachypleus tridentatus
Andronoctus australis
Centruroides sculpturatus
Chaerilus celebensis
Vietbocap lao
Bothriurus burmeisteri
Iurus dekanum
Superstinionia donensis
Hadrurus arizonensis
Scorpiops sp.
Liocheles australasiae
Pandinus imperator
Mastigoproctus giganteus
Damon sp.
Damon variegatus
Dysdera crocota
Pholcus manueli
Uloborus glomosus
Amaurobius ferox
Pisaurina mira
Oecobius cellariorum
Microdipoena guttata
Ero leonina
Neoscona arabesca
Antrodiaetus unicolor
Megahexura fulva
Sophodros rufipes
Hypochilus pococki
Liphistius malayanus
Microhexura montivaga
Brachythele longitarsus
Aphonopelma iviei
Paratropis sp.
Pionothele sp.
96
51
79
80
99
59
97
54
99
88
83
83
63
63
65
74
74
74
0.1 substitutions per site
Onychophora
Myriapoda
Pancrustacea
Pycnogonida
Parasitiformes
Acariformes
Pseudoscorpiones
Solifugae
Ricinulei
Opiliones
Xiphosura
Scorpiones
Uropygi + Amblypygi
Araneae
Arthropoda
Chelicerata
Euchelicerata
Acari
Arachnopulmonata
Tetrapulmonata
b

Supplementary Figure 2. Phylogenetic trees derived from the ML analysis of Matrix D. **(A)** Phylogeny recovered from the ML analysis of Matrix D. The tree obtained from this analysis is in excellent agreement with those obtained from our other ML analyses except that the five species with the worst quality transcriptomes (*Endeis spinosa*, *Anoplodactylus eroticus*, *Epiperipatus* sp., *Euperipatoides kanangrensis* and *Euphrynichus bacillifer*) formed a clearly artificial group nested inside Solifugae. **(B)** A reanalysis of this dataset that excluded the five species with the worst transcriptomes (*Endeis spinosa*, *Anoplodactylus eroticus*, *Epiperipatus* sp., *Euperipatoides kanangrensis* and *Euphrynichus bacillifer*) recovered a topology in excellent agreement with that from the analysis of Matrix B. In particular monophyletic Acari is recovered irrespective of whether the five species with poor quality transcriptomes are included or excluded. We conclude that neither the size of the matrix nor the trimming strategy impacts significantly on the retrieved topology.

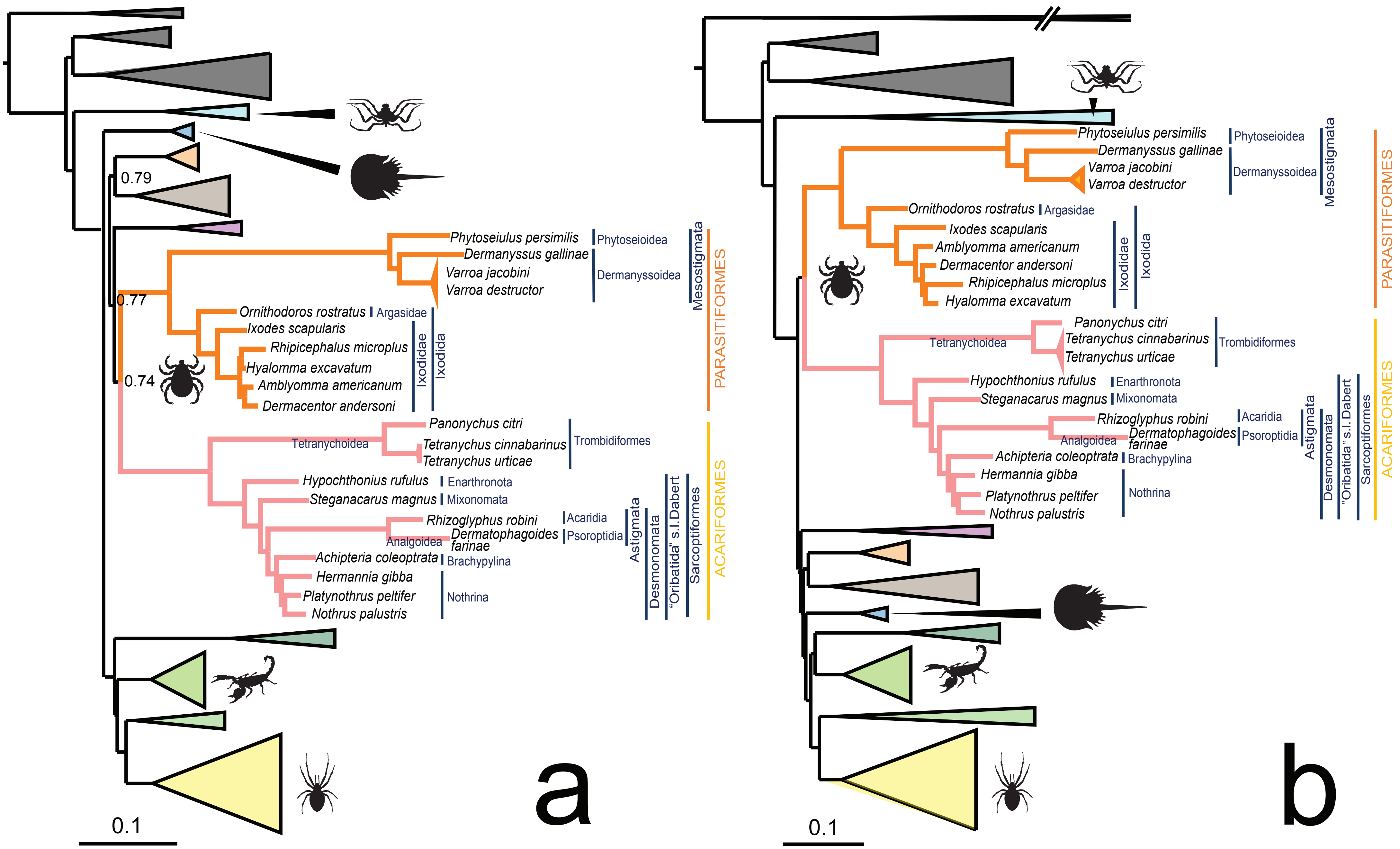
0.79
0.77
0.74
Phytoseiulus persimilis
Dermanyssus gallinae
Varroa jacobini
Varroa destructor
Ornithodoros rostratus
Ixodes scapularis
Rhipicephalus microplus
Hyalomma excavatum
Amblyomma americanum
Dermacentor andersoni
Panonychus citri
Tetranychus cinnabarinus
Tetranychus urticae
Hypochthonius rufulus
Steganacarus magnus
Rhizoglyphus robini
Dermatophagoides farinae
Achipteria coleoptrata
Hermannia gibba
Platynothrus peltifer
Nothrus palustris
Phytoseioidea
Dermanyssoidea
Mesostigmata
Argasidae
Ixodidae
Ixodida
PARASITIFORMES
Tetranychoidea
Trombidiformes
Enarthronota
Mixonomata
Acaridia
Psoroptidia
Analgoidea
Brachypylina
Nothrina
Astigmata
Desmonomata
"Oribatida" s.l. Dabert
Sarcoptiformes
ACARIFORMES
0.1
a
b

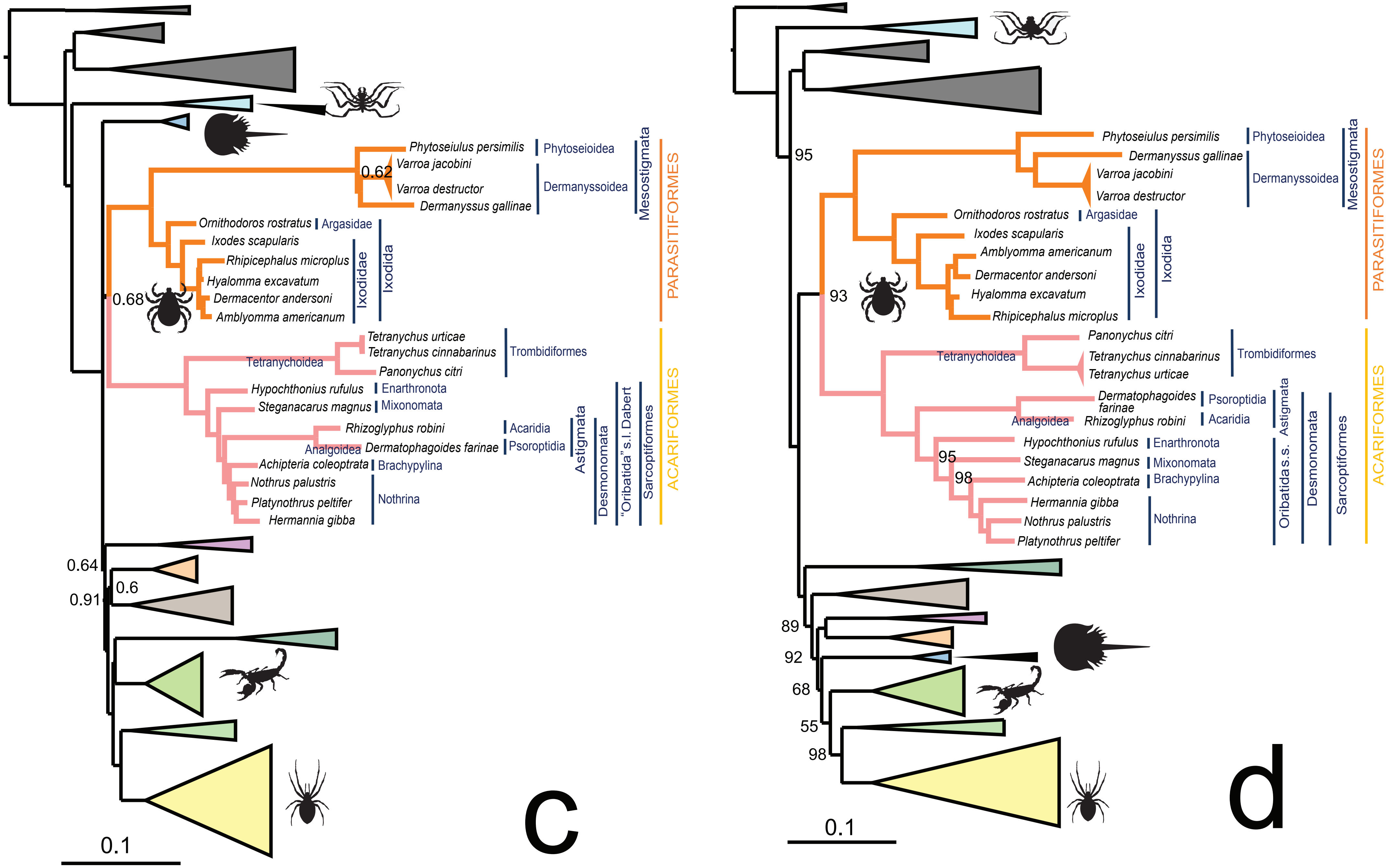
Phytoseiulus persimilis
Varroa jacobini
Varroa destructor
Dermanyssus gallinae
Phytoseioidea
Dermanyssoidea
Mesostigmata
PARASITIFORMES
0.62
Ornithodoros rostratus
Argasidae
Ixodes scapularis
Rhipicephalus microplus
Hyalomma excavatum
Dermacentor andersoni
Amblyomma americanum
Ixodidae
Ixodida
0.68
Tetranychus urticae
Tetranychus cinnabarinus
Panonychus citri
Tetranychoidea
Trombidiformes
Hypochthonius rufulus
Enarthronota
Steganacarus magnus
Mixonomata
Rhizoglyphus robini
Acaridia
Analgoidea
Dermatophagoides farinae
Psoroptidia
Achipteria coleoptrata
Brachypylina
Nothrus palustris
Platynothrus peltifer
Hermannia gibba
Nothrina
Astigmata
Desmonomata
"Oribatida" s.l. Dabert
Sarcoptiformes
ACARIFORMES
0.64
0.6
0.91
0.1
c
95
Phytoseiulus persimilis
Phytoseioidea
Dermanyssus gallinae
Varroa jacobini
Varroa destructor
Dermanyssoidea
Mesostigmata
PARASITIFORMES
Ornithodoros rostratus
Argasidae
Ixodes scapularis
Amblyomma americanum
Dermacentor andersoni
Hyalomma excavatum
Rhipicephalus microplus
Ixodidae
Ixodida
93
Panonychus citri
Tetranychoidea
Tetranychus cinnabarinus
Tetranychus urticae
Trombidiformes
Dermatophagoides farinae
Psoroptidia
Analgoidea
Rhizoglyphus robini
Acaridia
Astigmata
Hypochthonius rufulus
Enarthronota
95
Steganacarus magnus
Mixonomata
98
Achipteria coleoptrata
Brachypylina
Hermannia gibba
Nothrus palustris
Platynothrus peltifer
Nothrina
Oribatida s.s.
Desmonomata
Sarcoptiformes
ACARIFORMES
89
92
68
55
98
0.1
d

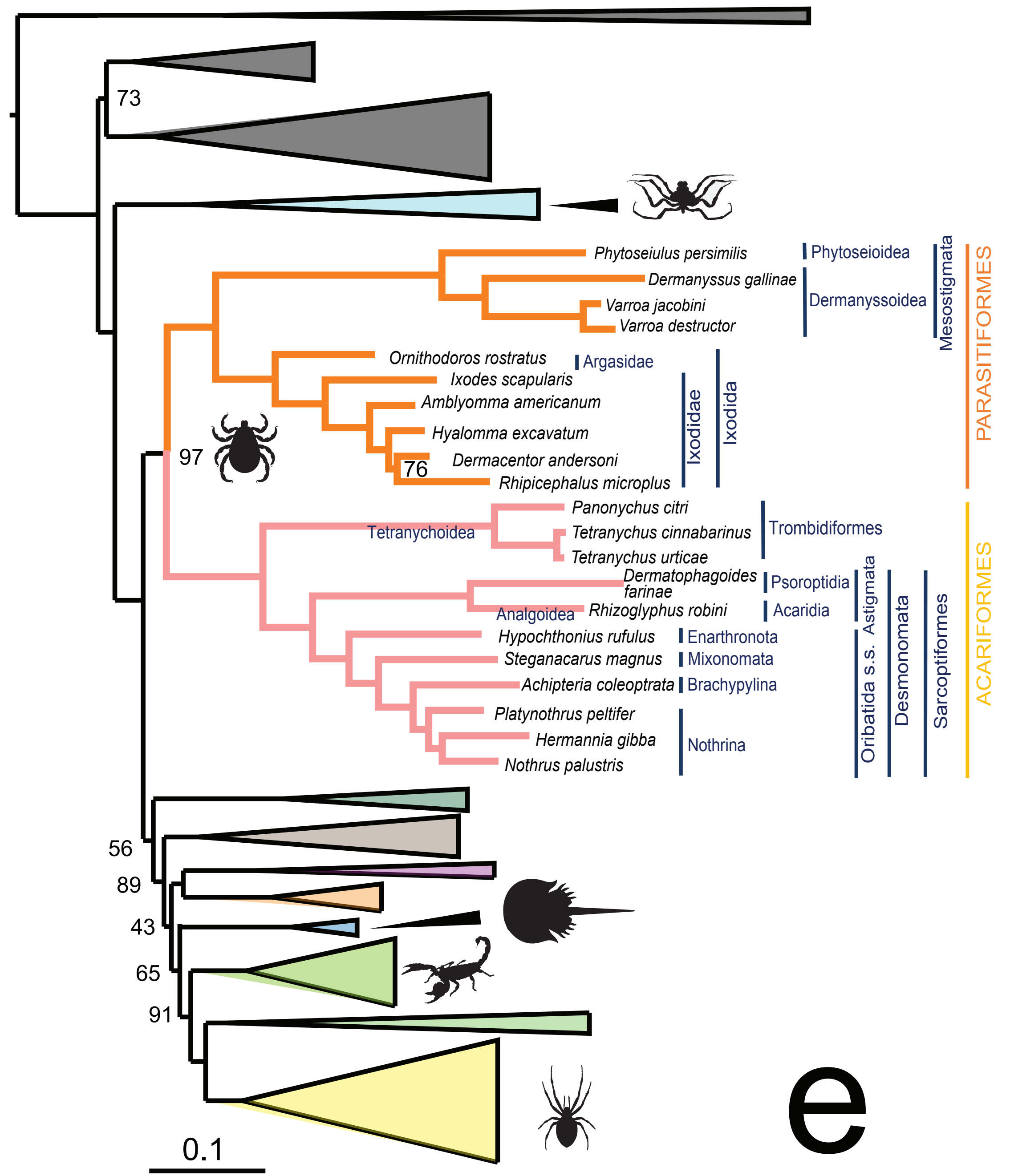

Supplementary Figure 3. Schematic representation of the Bayesian and ML phylogenies at the order level with focus on the internal clades of Acari. **(A)** Schematic representation of the results of the CAT-GTR+G analysis of Matrix A.**(B)** Schematic representation of the results of the CAT-GTR+G analysis of Matrix B. **(C)** Schematic representation of the results of the CAT-GTR +G analysis of Matrix A, after Dayhoff-6 recoding **(D)** Schematic representation of the relationships inferred from Matrix A using ML under the LG+I+G4 model. **(E)** Schematic representation of the relationships inferred from Matrix B using ML under the LG+I+G4 model. Support values represent posterior probabilities in A, B and C, and bootstrap values in D and E, with lack of a number indicating maximum support. Convergence statistics for (A) Burnin = 3,000, Total Cycles = 10,000, subsampling frequency = 20, Maxdif = 0.87, Minimal effective size = 23, (B) Burnin = 3,000, Total Cycles = 10,000, subsampling frequency = 20, Maxdif = 0.07, Minimal effective size = 32 and (C) Burnin = 3,000, Total Cycles = 12,000, subsampling frequency = 20, Maxdif = 0.08; Minimal effective size = 72. Silhouettes have been designed by ART.

Supplementary Table 1. List of all species included in the study with NCBI accession numbers.

Order/Clade	Species	Source	Peptides prediction	Accession
Pycnogonida	*Endeis spinosa*	454	2930	LIBEST_025662
	***Pycnogonum* sp.**	Illumina	26668	SRR8745912
	Anoplodactylus eroticus	454	2595	Sharma *et al.* 2014 (generated on Dunn *et al.* 2008)
Xiphosura	***Limulus polyphemus***	Illumina	17824	SRR1145732
	Tachypleus tridentatus	Illumina	47095	SRR946952
	Carcinoscorpius rotundicauda	Illuimina	82789	SRR1511637
Acariformes	***Tetranychus urticae***	Illumina	26106	PRJEB6152
	Dermatophagoides farinae	Illumina	36507	SRR1016494
	Panonychus citri	Illumina	24400	SRR341928
	Rhizoglyphus robini	Illumina	72646	PRJNA213807
	Steganacarus magnus	Illumina	60104	SRR4039729
	Hypochthonius rufulus	Illumina	29268	SRR4039020
	Platynothrus peltifer	Illumina	42123	SRR4039728
	Achipteria coleoptrata	Illumina	54612	SRR4039018
	Hermannia gibba	Illumina	64632	SRR4039019
	Nothrus palustris	Illumina	57025	SRR4039021
	Tetranychus cinnabarinus	Illumina	23425	SRR519097
Parasitiformes	*Amblyomma americanum*	Illumina	32598	PRJNA238773
	Dermacentor andersoni	Illumina	30501	PRJNA238802
	Ixodes scapularis	Illumina	56503	SRR1189647
	Rhipicephalus microplus	Illumina	13004	SRR1186998
	Hyalomma excavatum	Illumina	54671	PRJNA311286
	Ornithodoros rostratus	Illumina	37109	PRJNA270484
	Dermanyssus gallinae	454	39197	SRR658515
	Varroa destructor	Illumina	16085	SRR3927486
	Varroa jacobini	Illumina	12342	SRR3634772
	Phytoseiulus persimilis	454	21218	DRR001717
Ricinulei	*Pseudocellus* sp. (pearsei)	Illumina GAII	5922	SRR1146686
	Ricinoides atewa	Illumina GAII	14324	SRR1145743
	Cryptocellus sp. n. RF-2015	Illumina	49645	SRR1982218
	Ricinoides karschii	Illumina	87143	SRR1972991
	Cryptocellus becki	Illumina	128981	SRR1979416
Solifugae	***Eremobates* sp.**	Illumina GAII	11,765	SRR1146672
	Gluvia dorsalis	454	5404	SRR1141096
	Galeodes sp.	Illumina	422228	SRR8745910
Pseudoscorpiones	***Synsphyronus apimelus***	Illumina	17820	SRR1145733
	Haplochernes kraepelini	Illumina	16376	SRR1767661
	Hesperochernes sp.	Illumina	30903	SRR1514877
	Neobisium carcinoides	Illumina	24142	SRR7293662
Scorpiones	*Bothriurus burmeisteri*	Illumina	20574	SRR1721670
	Chaerilus celebensis	Illumina	24310	SRR1721804
	Centruroides sculpturatus	Illumina	16440	SRR1515193
	Hadrurus arizonensis	Illumina	19266	SRR1721733
	Iurus dekanum	Illumina	17619	SRR1721734
	Liocheles australasiae	Illumina	22581	SRR1721664
	Superstitionia donensis	Illumina	23916	SRR1721951
	Vietbocap lao	Illumina	20007	SRR1721740

	Androctonus australis	Illumina	19170	SRR1724216
	Pandinus imperator	Illumina	20279	SRR1721600
	Scorpiops sp.	Illumina	24941	SRR1721951
Uropygi	***Mastigoproctus giganteus***	Illumina GAII	17674	SRR1145698
Amblypygi	***Damon variegatus***	Illumina GAII	11823	SRR1145694
	Euphrynichus bacillifer	454	3895	SRR1141095
	Damon sp.	Illumina	24564	SRR8745909
Opiliones	*Larifuga capensis*	Illumina	10506	SRR1145742
	Metasiro americanus	Illumina GAII	16556	SRR618563
	Pachylicus acutus	Illumina	14202	SRR1146670
	Protolophus singularis	Illumina	13987	SRR1145700
	Trogulus martensi	Illumina	12765	SRR1145730
	Hesperonemastoma modestum	Illumina	8845	SRR1145728
	Siro boyerae	Illumina	11387	SRR1145699
Araneae	***Liphistius malayanus***	Illumina	11221	SRR1145736
	Neoscona arabesca	Illumina	16594	SRR1145741
	Dysdera crocota	Illumina	30336	SRR1328258
	Pholcus manueli	Illumina	27519	SRR1365208
	Oecobius cellariorum	Illumina	30394	SRR1365089
	Uloborus glomosus	Illumina	15941	SRR1328334
	Amaurobius ferox	Illumina	19707	SRR1329250
	Pisaurina mira	Illumina	15940	SRR1365651
	Microdipoena guttata	Illumina	17704	SRR1333842
	Sphodros rufipes	Illumina	23297	SRR1514908
	Antrodiaetus unicolor	Illumina	20709	SRR1514897
	Megahexura fulva	Illumina	30559	SRR1514891
	Microhexura montivaga	Illumina	17718	SRR1514890
	Brachythele longitarsus	Illumina	19334	SRR1514875
	Pionothele n. sp.	Illumina	10538	SRR1514906
	Paratropis sp.	Illumina	9961	SRR1514893
	Aphonopelma iviei	Illumina	11968	SRR1514871
	Hypochilus pococki	Illumina	29268	SRR1514889
	Ero leonina	Illumina	33289	SRR1514886
Onychophora	***Peripatopsis overbergiensis***	Illumina	12846	SRX451023
Onychophora	*Euperipatoides kanangrensis*	EST	3267	Dunn et al. 2008
Onychopohra	*Epiperipatus sp. TB-2001*	EST	514	Roeding, F., *et al.* 2007
Crustacea	*Argulus siamensis*	Illumina	48641	SRR514120
Crustacea	*Gammarus chevreuxi*	Illumina	28997	GFCV00000000.1
Crustacea	*Calanus finmarchicus*	Illumina	126873	SRR1153469
Crustacea	***Daphnia pulex***	WGS Sanger	30611	PRJNA12756
Hexapoda	*Orchesella cincta*	WGS	20257	PRJNA294050
Hexapoda	*Drosophila melanogaster*	WGS	30443	PRJNA13812
Hexapoda	*Blattella germanica*	454	7302	PRJNA248247
Hexapoda	***Tribolium castaneum***	WGS	18534	PRJNA12540
Myriapoda	*Alipes grandidieri*	Illumina	18814	SRR619311
Myriapoda	***Scutigera coleoptrata***	Illumina	31758	SRR1158078
Myriapoda	*Hanseniella* sp.	Illumina	50853	SRR3458647
Myriapoda	*Glomeris marginata*	Illumina	66936	SRR3458641

In black species used initially during the OMA orthology-assignment

Supplementary Table 2. List of orthologs shared between Matrix A and B.

BLAST genes	OMA equivalent OrthoGroup	BLAST genes	OMA equivalent OrthoGroup
ar21	OG12060	*mcmD*	OG1048
arp23	OG3395	*nsf1C*	OG2299
atpsynthalphaamt	OG1215	*nsf1G*	OG1789
cctA	OG1188	*nsf1J*	OG2110
cctB	OG1606	*nsf1K*	OG1788
cctD	OG1685	*nsf1L*	OG1954
cctE	OG1258	*nsf1M*	OG1423
cctG	OG1666	*nsf2A*	OG307
cctN	OG1544	*orf2*	OG12179
cctZ	OG1591	*ornamtransa*	OG3188
cpn60mt	OG1263	*psmaA*	OG7137
crfg	OG1895	*psmaB*	OG6270
ef1EF1	OG1279	*psmaC*	OG6195
ef1RF3	OG1393	*psmaD*	OG6991
ef2EF2	OG277	*psmaE*	OG6938
eif5a	OG12223	*psmaF*	OG7384
fibri	OG5753	*psmaG*	OG7144
fpps	OG13935	*psmbH*	OG13150
g122	OG24237	*psmbI*	OG10010
g127	OG30226	*psmbJ*	OG11178
g46	OG4754	*psmbK*	OG8076
g70	OG26595	*psmbL*	OG11980
g78	OG6693	*psmbM*	OG7815
g7	OG29912	*psmbN*	OG12279
grc5	OG7044	*pyrdehydroe1bmt*	OG4622
hsp70E	OG791	*rad23*	OG7719
hsp70mt	OG827	*rf1*	OG1348
hsp90C	OG518	*rla2A*	OG30868
hsp90E	OG577	*rla2B*	OG28197
if1a	OG13352	*rpl11b*	OG9660
if2b	OG5408	*rpl12b*	OG13588
if2g	OG1506	*rpl13*	OG8643
if4aa	OG2006	*rpl14a*	OG17699
if4ab	OG1753	*rpl15a*	OG8245
if6	OG7623	*rpl16b*	OG9413
l12eA	OG16557	*rpl17*	OG9435
l12eC	OG19114	*rpl18*	OG10858
l12eD	OG5608	*rpl19a*	OG8244

BLAST genes	OMA equivalent OrthoGroup
rpl1	OG7857
rpl20	OG10345
rpl21	OG12888
rpl22	OG18472
rpl23a	OG14123
rpl24A	OG14344
rpl24B	OG19045
rpl25	OG14181
rpl26	OG13968
rpl27	OG15207
rpl2	OG5237
rpl30	OG20031
rpl31	OG18135
rpl32	OG15164
rpl33a	OG17810
rpl35	OG20637
rpl36	OG21070
rpl37a	OG22673
rpl3	OG1953
rpl42	OG19715
rpl43b	OG24650
rpl4B	OG2935
rpl6	OG8371
rpl7A	OG7413
rpl9	OG10508
rpp0	OG4543
rps10	OG16379
rps11	OG12202
rps13a	OG12931
rps14	OG12579
rps15	OG13130
rps16	OG14312
rps17	OG16498
rps18	OG12247
rps19	OG14901
rps1	OG5421
rps20	OG20114
rps22a	OG15319
rps23	OG12881

BLAST genes	OMA equivalent OrthoGroup
rps24	OG17433
rps25	OG20215
rps26	OG18162
rps27a	OG15504
rps2	OG5265
rps3	OG6317
rps4	OG4960
rps5	OG7696
rps6	OG5777
rps7	OG9651
rps8	OG7796
rps9	OG9104
sadhchydrolaseE1	OG2304
sap40	OG6543
srp54	OG1177
srs	OG2547
stbproptase2ab	OG3204
stcproptase2ac	OG3672
suca	OG5310
tif2a	OG5313
tribe1009	OG6949
tribe1015	OG6355
tribe1026	OG7993
tribe1047	OG12765
tribe1050	OG29425
tribe1054	OG13294
tribe1081	OG7978
tribe1099	OG22457
tribe1118	OG6174
tribe1132	OG22480
tribe1136	OG27806
tribe1169	OG12849
tribe1170	OG28247
tribe1181	OG12369
tribe1190	OG23564
tribe1200	OG28566
tribe1204	OG24213
tribe1244	OG9939
tribe1245	OG15596

BLAST genes	OMA equivalent OrthoGroup
tribe1294	OG13896
tribe1335	OG12455
tribe1378	OG11886
tribe1381A	OG5055
tribe1409	OG26511
tribe281	OG23080
tribe333	OG22471
tribe495	OG7782
tribe532	OG16260
tribe542	OG19046
tribe550	OG18450
tribe572	OG12562
tribe585A	OG12661
tribe586	OG12786
tribe613	OG37992
tribe622	OG5261
tribe629A	OG18880
tribe646	OG31788
tribe647	OG14630
tribe683	OG29404
tribe700	OG17191
tribe716	OG16597
tribe717	OG29212
tribe739	OG36538
tribe740	OG27048
tribe756	OG6415
tribe764	OG20345
tribe768	OG19978
tribe801mt	OG10726
tribe831	OG8814
tribe832	OG5662
tribe850	OG12417
tribe852	OG21157
tribe893	OG17091
tribe895	OG12638
tribe896	OG10414
tribe905	OG34217
tribe906	OG17038
tribe927	OG32840

BLAST genes	OMA equivalent OrthoGroup
tribe930	OG18359
tribe942	OG10200
u2snrnp	OG7268
vacaatpasepl21a	OG12244
vata	OG845
vatb	OG1247
vatc	OG3579
vate	OG11672
vatpased	OG10153
vdac2	OG7309
w09c	OG6215

Supplementary Table 3: Comparison between OMA and the BLAST-based approach orthology assignments. Green represents exact match between both methods, orange represents ortholog presence in one method but not in the other, turquoise represents absence of orthologs in both methods, and magenta represents disagreement between methods.

High-occupancy genes

**Species**	*BLAST ef1EF1*	OMA OG1279	*BLAST ef1EF3*	OMA OG1393	*BLAST ef2EF2*	OMA OG277	*BLAST if4aa*	OMA OG2006	*BLAST nsf1G.fas*	OMA OG1789	*BLAST nsf1J*	OMA OG2110	*BLAST nsf1K*	OMA OG1788	*BLAST nsf1L*	OMA OG1954	*BLAST nsf1M*	OMA OG1423	*BLAST nsf2A*	OMA OG307
Damon variegatus	=	=	/	/	X		X		=	=	X		X		X		=	=	=	=
Daphnia pulex	=	=	=	=		X	=	=	=	=	=	=	=	=	=	=	=	=	=	=
Dermatophagoides farinae	=	=	=	=	=	=	X		=	=	X		=	=	=	=	=	=	=	=
Dysdera crocota	=	=	=	=	X			X	X		=	=	=	=	=	=	=	=	=	=
Eremobates sp.	=	=	=	=	X		=	=	=	=	=	=	X		=	=	=	=	X	
Hypochthonius rufulus	=	=	X		=	=	=	=	=	=	=	=	=	=	=	=	=	=	=	=
Limulus polyphemus	=	=	=	=	=	=	=	=	=	=	=	=	=	=	=	=	=	=	=	=
Liocheles australasiae	=	=	X		=	=	X		=	=	=	=	X		=	=	=	=	X	
Liphistius malayanus	=	=	=	=	=	=	=	=	=	=	=	=	=	=	=	=	=	=	=	=
Mastigoproctus giganteus	=	=	=	=	=	=	=	=	=	=	=	=	=	=	=	=	X		=	=
Ornithodoros rostratus	=	=	=	=	=	=	=	=	=	=	=	=	=	=	=	=	=	=	=	=
Peripatopsis overbergiensis	=	=		X	=	=	=	=	X		X		=	=	X		=	=	X	
Pycnogonum sp.	X	X	=	=		X	=	=	=	=	=	=	=	=	=	=	=	=	=	=
Ricinoides atewa	=	=	X		X		X		=	=	=	=	X		X		=	=	=	=
Scutigera coleoptrata	=	=	=	=	=	=	=	=	=	=	=	=	X		=	=	=	=	=	=
Siro boyerae	=	=	=	=	=	=	=	=	=	=	=	=	=	=	=	=	=	=	=	=
Sphodros rufipes	=	=	=	=	=	=	=	=	=	=	=	=	=	=	=	=	=	=	=	=
Synsphyronus apimelus	X	X	=	=	X		=	=	=	=	=	=		X	=	=	=	=	=	=
Tetranychus urticae	=	=	=	=	=	=	=	=	=	=	=	=	=	=	X		=	=	X	
Tribolium castaneum	=	=	=	=	=	=	=	=	=	=	=	=	=	=	=	=	=	=	=	=
Trogulus martensi	=	=	=	=	=	=	=	=	=	=	=	=	=	=	=	=	=	=	=	=
Varroa destructor	=	=	X		=	=	X		=	=	=	=	=	=	=	=	=	=	=	=
Vietbocap lao	=	=	=	=	=	=	=	=	=	=	=	=	=	=	X		=	=	=	=

Single absence	39
Both absence (/)	2
Single presence (X)	39
Both presence (=)	376
Both presence different gen	4
Total	460

82% shared presence out of total possibilties
82% shared presence/absence
17% of genes are present in one matrix and not in the other
0.8% (2 cases out of 230) pair of orthologs were different

Low-occupancy genes

Species	*BLAST* *rpl43b*	OMA OG24650	*BLAST* *tribe646*	OMA OG18880	*BLAST* *tribe740*	OMA OG27048	*BLAST* *fpps*	OMA OG13935	*BLAST* *rpl37a*	OMA OG22673
Damon variegatus	/	/	=	=	/	/	X		/	/
Daphnia pulex	=	=	/	/	=	=	=	=	=	=
Dermatophagoides farinae	/	/	X		=	=	=	=	=	=
Dysdera crocota	/	/	X		=	=	X		=	=
Eremobates sp.	/	/	/	/	/	/	X		/	/
Hypochthonius rufulus		X	X		=	=	=	=	/	/
Limulus polyphemus	/	/	=	=	/	/	=	=	=	=
Liocheles australasiae	/	/	=	=	/	/	X		=	=
Liphistius malayanus	/	/	=	=	/	/	=	=	=	=
Mastigoproctus giganteus	/	/	=	=	/	/	X		=	=
Ornithodoros rostratus	X		X		=	=	=	=	=	=
Peripatopsis overbergiensis		X	X		/	/	X		X	
Pycnogonum sp.	/	/	X		/	/	=	=	=	=
Ricinoides atewa	/	/	=	=	/	/	X		/	/
Scutigera coleoptrata	X		X		=	=	/	/	=	=
Siro boyerae	X		=	=	/	/	/	/	/	/
Sphodros rufipes	=	=	X		=	=	=	=	=	=
Synsphyronus apimelus	/	/	=	=	/	/	=	=	/	/
Tetranychus urticae	/	/	X		=	=	=	=	/	/
Tribolium castaneum	/	/	=	=	=	=	X		=	=
Trogulus martensi	=	=	X		/	/	=	=	=	=
Varroa destructor		X	=	=	=	=	X		/	/
Vietbocap lao	/	/	=	=	=	=	X		/	/

Species	*BLAST* *tribe739*	OMA OG36538	*BLAST* *tribe683*	OMA OG29404	*BLAST* *rps10*	OMA OG16379	*BLAST* *tribe852*	OMA OG21157	*BLAST* *tribe1378*	OMA OG11886
Damon variegatus	X			X	=	=	=	=	X	
Daphnia pulex	=	=	=	=	/	/		X	X	
Dermatophagoides farinae	=	=	X		X		=	=	X	
Dysdera crocota	=	=	/	/	=	=	X		X	
Eremobates sp.	X		X		=	=	=	=	/	/
Hypochthonius rufulus	X			X	X		X		=	=
Limulus polyphemus	X			X	=	=	X		/	/
Liocheles australasiae	X		/	/	=	=	=	=	=	=
Liphistius malayanus	=	=	=	=	=	=	=	=	=	=
Mastigoproctus giganteus	X			X	=	=	=	=	X	
Ornithodoros rostratus	=	=	/	/	=	=	=	=	=	=
Peripatopsis overbergiensis	=	=	=	=	X		=	=	=	=
Pycnogonum sp.	=	=		X	X			X	=	=
Ricinoides atewa	X			X	=	=	=	=	/	/
Scutigera coleoptrata	=	=	=	=	=	=	=	=	=	=
Siro boyerae		X	/	/	=	=	=	=	=	=
Sphodros rufipes	=	=	/	/	X		X		=	=
Synsphyronus apimelus	=	=		X	X		X		=	=
Tetranychus urticae	X		/	/	X		/	/	=	=
Tribolium castaneum	=	=	=	=	=	=	=	=	=	=
Trogulus martensi	X		/	/	=	=	/	/	=	=
Varroa destructor	=	=		X	=	=	X		X	
Vietbocap lao	=	=	/	/	=	=	=	=	=	=

Single absence	68
Both absence (/)	106
Single presence (X)	68
Both presence (=)	218
Total	460

47% shared presence out of total possibilties
70.4% shared presence/absence
30% of genes are present in one matrix and not in the other
