# Supplementary material for: Increasing species sampling in chelicerate genomic-scale datasets provides support for monophyly of Acari and Arachnida
Source: Nat Commun. 2019 May 24;10:2295. doi: 10.1038/s41467-019-10244-7 (PMC6534568; doi:10.1038/s41467-019-10244-7)
Supplement: Supplementary file 3 — Reporting Summary [file 41467_2019_10244_MOESM3_ESM.pdf]

nature research

Corresponding author(s): Davide Pisani

Last updated by author(s): Mar 20, 2019

Reporting Summary

Nature Research wishes to improve the reproducibility of the work that we publish. This form provides structure for consistency and transparency in reporting. For further information on Nature Research policies, see Authors & Referees and the Editorial Policy Checklist.

Statistics

For all statistical analyses, confirm that the following items are present in the figure legend, table legend, main text, or Methods section.

n/a	Confirmed	
☐	☒	The exact sample size (*n*) for each experimental group/condition, given as a discrete number and unit of measurement
☒	☐	A statement on whether measurements were taken from distinct samples or whether the same sample was measured repeatedly
☒	☐	The statistical test(s) used AND whether they are one- or two-sided<br>*Only common tests should be described solely by name; describe more complex techniques in the Methods section.*
☒	☐	A description of all covariates tested
☒	☐	A description of any assumptions or corrections, such as tests of normality and adjustment for multiple comparisons
☒	☐	A full description of the statistical parameters including central tendency (e.g. means) or other basic estimates (e.g. regression coefficient) AND variation (e.g. standard deviation) or associated estimates of uncertainty (e.g. confidence intervals)
☒	☐	For null hypothesis testing, the test statistic (e.g. *F*, *t*, *r*) with confidence intervals, effect sizes, degrees of freedom and *P* value noted<br>*Give P values as exact values whenever suitable.*
☐	☒	For Bayesian analysis, information on the choice of priors and Markov chain Monte Carlo settings
☒	☐	For hierarchical and complex designs, identification of the appropriate level for tests and full reporting of outcomes
☒	☐	Estimates of effect sizes (e.g. Cohen's *d*, Pearson's *r*), indicating how they were calculated

Our web collection on statistics for biologists contains articles on many of the points above.

Software and code

Policy information about availability of computer code

Data collection	https://www.ncbi.nlm.nih.gov
Data analysis	Trinity version 2.0.3<br>Trimmomatic<br>cd-hit-v4.6.4<br>TransDecoder-3.0.0<br>BLAST<br>www.github.com/jairly/MoSuMa_tools/<br>MUSCLE v3.7<br>IQTree-1.6.2<br>ModelFinder<br>SequenceMatrix v100<br>GBlocks v0.91b<br>OMA.2.0.0<br>trimAl v1.4<br>APE 5.0<br>PAUP4.0a<br>PhyloBayes MPI v1.5a

For manuscripts utilizing custom algorithms or software that are central to the research but not yet described in published literature, software must be made available to editors/reviewers. We strongly encourage code deposition in a community repository (e.g. GitHub). See the Nature Research guidelines for submitting code & software for further information.

Data

Policy information about availability of data

All manuscripts must include a data availability statement. This statement should provide the following information, where applicable:

- Accession codes, unique identifiers, or web links for publicly available datasets
- A list of figures that have associated raw data
- A description of any restrictions on data availability

All scripts, individual gene alignments, amino acid matrices and phylogenetic trees have been deposited as a Bitbucket repository and can be accessed at https://bitbucket.org/bzxdp/lozano_fernandez_2019 . The transcriptomes generated as part of our study are available at NCBI Sequence Read Archive – BioProject PRJNA438779 [https://www.ncbi.nlm.nih.gov/bioproject/PRJNA438779]. Individual SRA numbers for the raw read data of each species are listed in Supplementary Table 1.

Field-specific reporting

Please select the one below that is the best fit for your research. If you are not sure, read the appropriate sections before making your selection.

☐ Life sciences ☐ Behavioural & social sciences ☒ Ecological, evolutionary & environmental sciences

For a reference copy of the document with all sections, see nature.com/documents/nr-reporting-summary-flat.pdf

Ecological, evolutionary & environmental sciences study design

All studies must disclose on these points even when the disclosure is negative.

Study description	We present a phylogenomic investigation of Chelicerata, utilising both new and more complete sequence information and a robust inferential methodology.
Research sample	We compiled molecular datasets based on transcriptomic data from 95 species, mostly from Illumina transcriptomes that were largely retrieved from public repositories (NCBI), and four species that we newly sequenced. The taxonomic sample spanned 80 chelicerates, 21 of them representing Acari, and 15 outgroups. Our dataset includes representatives of all arachnid orders, with the exception of two minor lineages, Palpigradi and Schizomida, for which genomic-scale data are still missing. We include sequence information from three of four living horseshoe crab species, and most importantly we expand taxon sampling within mites and ticks, including short-branched representatives of Sarcoptiformes (Acariformes) and Mesostigmata (Parasitiformes).
Sampling strategy	We prioritized maximizing the amount of diversity within the group, trying to have en even representation of chelicerates at the order level, and particularly focusing on increasing the representation of lineages that that are prone to the effects of Long Branch Attraction, like Acariformes, Parasitiformes or Pseudoscorpiones.
Data collection	Molecular data was largely retrieved from public repositories, such NCBI. For those four newly generated transcriptomes, they were generated at the University of Maynooth and assembled by Robert Carton.
Timing and spatial scale	Transcriptomic and genomic samples were retrieved from public repositories during 2015 and 2016. We generated the four Illumina transcriptomes during the 2011 to 2013 period.
Data exclusions	No data were excluded from the analyses
Reproducibility	For the Bayesian analyses, we ran two chains until convergence. We make available all generated data so others researchers can reproduce our experiments.
Randomization	Randomization was not relevant to our study. As we have tried to maximize the inclusion of a wide diversity of chelicerates (some orders in which there are just available a few transcriptomes), performing experiments removing some of this data would have possibly exacerbated systematic errors, such as long branch attraction.
Blinding	Blinding is not relevant to this kind of study.

Did the study involve field work? ☐ Yes ☒ No

Reporting for specific materials, systems and methods

We require information from authors about some types of materials, experimental systems and methods used in many studies. Here, indicate whether each material, system or method listed is relevant to your study. If you are not sure if a list item applies to your research, read the appropriate section before selecting a response.

Materials & experimental systems

n/a	Involved in the study
☒	☐ Antibodies
☒	☐ Eukaryotic cell lines
☒	☐ Palaeontology
☐	☒ Animals and other organisms
☒	☐ Human research participants
☒	☐ Clinical data

Methods

n/a	Involved in the study
☒	☐ ChIP-seq
☒	☐ Flow cytometry
☒	☐ MRI-based neuroimaging

Animals and other organisms

Policy information about studies involving animals; ARRIVE guidelines recommended for reporting animal research

Laboratory animals	The study did not involve laboratory animals.
Wild animals	The study involved the collection of small arthropods in ocean or terrestrial environments. The specimens were collected and determined at the genus or species level by collaborators, and stored in RNA later for posterior RNA extraction.
Field-collected samples	We generated four new transcriptomes: the sea spider Pycnogonum sp., the solifugid Galeodes sp., the pseudoscorpion Neobisium carcinoides, and the amblypygid Damon sp. The specimens were collected and determined at the genus or species level by collaborators, and stored in RNA later for posterior RNA extraction. We have complied with all relevant ethical regulations for animal testing while collecting and processing these animals.
Ethics oversight	No ethical approval was required for the handling of small arthropods.

Note that full information on the approval of the study protocol must also be provided in the manuscript.
